# Pathology Foundation Models

**DOI:** 10.31662/jmaj.2024-0206

**Published:** 2024-12-20

**Authors:** Mieko Ochi, Daisuke Komura, Shumpei Ishikawa

**Affiliations:** 1 Department of Preventive Medicine, Graduate School of Medicine, The University of Tokyo, Tokyo, Japan

**Keywords:** artificial intelligence, medicine, pathology, foundation model, generalist medical AI

## Abstract

Pathology plays a crucial role in diagnosing and evaluating patient tissue samples obtained via surgeries and biopsies. The advent of whole slide scanners and the development of deep learning technologies have considerably advanced this field, promoting extensive research and development in pathology artificial intelligence (AI). These advancements have contributed to reduced workload of pathologists and supported decision-making in treatment plans. Large-scale AI models, known as foundation models (FMs), are more accurate and applicable to various tasks than traditional AI. Such models have recently emerged and expanded their application scope in healthcare. Numerous FMs have been developed in pathology, with reported applications in various tasks, such as disease and rare cancer diagnoses, patient survival prognosis prediction, biomarker expression prediction, and scoring of the immunohistochemical expression intensity. However, several challenges persist in the clinical application of FMs, which healthcare professionals, as users, must be aware of. Research to address these challenges is ongoing. In the future, the development of generalist medical AI, which integrates pathology FMs with FMs from other medical domains, is expected to progress, effectively utilizing AI in real clinical settings to promote precision and personalized medicine.

## 1. Introduction for Digital Pathology

### (a) Development of digital pathology

For more than 50 years, surgical pathology has played a vital role in diagnosing diseases, evaluating disease progression, and elucidating causes by observing patient tissue sections obtained from surgeries and biopsies through formalin fixation, embedding, and staining. Next, these sections were examined by trained pathologists under microscopes. During this process, several scoring systems, such as the Gleason score for prostate cancer and the Nottingham score for breast cancer, were utilized to grade tumors based on their morphology. These scores provide essential information for determining treatment plans; however, given that they are determined based on the pathologist’s subjective judgment, a notable variability exists between pathologists. Moreover, some indicators impose a burden on pathologists in routine practice ^(1)^. However, introducing whole slide scanners in the 1990s easily created digital images of entire specimens with the same resolution as microscopes. This development highlighted the application of image analysis and machine learning technologies to histopathology, thereby expanding digital pathology, wherein the interpretation and analysis of digitized images (whole slide images [WSI]) can be performed quantitatively using computers. Furthermore, the remarkable speed of technological innovation in deep learning developed artificial intelligence (AI) that reduces the workload of pathologists and aids in predicting patient prognosis and supporting treatment decisions based on WSI, further resulting in research and development of AI with high potential clinical utility ^(2)^.

### (b) Current applications of AI in pathology

The applications of current pathology AI are broadly categorized into three types: i) improving the accuracy and efficiency of pathology diagnosis, ii) predicting patient prognosis and supporting treatment decisions, and iii) integrating data with genomic information.

Some items in ii) include iii) as elemental technology.

#### i) Improving the accuracy and efficiency of pathology diagnosis

AI enables accurate quantitative evaluation of various tissue features, such as immunohistochemical biomarker evaluation, cell counting, spatial cell arrangement, structural density, distribution patterns, and tissue structure, thereby developing diagnostic AI tools that diagnose with the same or higher accuracy obtained by general pathologists in specific tissues or provide information that is difficult to identify by pathologists. Examples include diagnostic support tools that evaluate the morphological features necessary for tumor assessment, such as tumor grade, histological type, and invasion extent, and automatic quantification tools for immunohistochemical staining intensity. For example, using tools, such as Mindpeak Breast Ki-67 RoI and Mindpeak ER/PR RoI, which automate the evaluation of Ki-67 and estrogen and progesterone receptors in breast cancer, interobserver agreement among pathologists increases ^(3)^. Additionally, AI has been developed to optimize the pathology workflow by triaging case priorities, categorizing cases based on priority, and checking specimen quality and identification ^(4), (5), (6)^.

#### ii) Predicting patient prognosis and supporting treatment decisions

After years of research, several morphological features of histopathological tissues, such as tumor grade and tissue subtypes, have been established and proven useful as indicators for predicting patient prognosis. Similarly, various clinical and genetic data, including treatment effects, responsiveness, resistance obtained from electronic medical records, and cancer gene panel test results, are crucial for predicting patient prognosis. The development of straightforward and accurate prognostic indicators has been reported using AI to effectively correlate and integrate these data with numerous histopathological findings obtained from pathology images, which have not yet been established as prognostic indicators. For example, Shi et al. developed a tumor microenvironment (TME) signature by automatically quantifying TME from pathological images of colorectal cancer, aiding in patient prognosis stratification ^(7)^. Reportedly, integrating individual histopathological features and, in some cases, clinical and genetic information, into a single classification system accurately reflects the nature and behavior of tumors. Pathology AI applied to prognosis prediction is expected to contribute to appropriate treatment selection and stratification of patients, promoting precision and personalized medicine ^(1), (2), (8)^.

#### iii) Integrating data with genomic information

The research and development of AI associating the phenotypes of histopathological tissue morphology with genomic profiles is attracting considerable attention. For example, Jaume et al. developed biologically and histopathologically interpretable AI that integrates genomic information and WSI using large datasets, such as the TCGA ^(9)^ with WSI and bulk transcriptome data ^(10)^. This research is essential for understanding the biological mechanisms underlying cancer and selecting targeted therapies. Furthermore, studies estimating gene mutations and protein expression levels from pathology images to reduce the delay in patient treatment initiation because of the weeks-long time required for clinicians to obtain genetic and immunological test results have been conducted ^(11), (12)^. Such research is progressing concerning developing AI that integrates multimodal information and presents it in an interpretable form for pathologists and clinicians.

## 2. Introduction for Foundation Models

### (a) Development of foundation models

Large-scale AI models called foundation models (FMs) have emerged ^(13)^ with 1) advancing social networking services, 2) the global spread of COVID-19, which increased the amount of digital data, 3) improved computational efficiency owing to hardware advancements, and 4) development of new AI architectures such as neural networks and transformers. FMs are applicable to a wider range of tasks than traditional AI models. A human-centered AI institute at Stanford defined FM as “a foundation model is any model that is trained on broad data (generally using self-supervision at scale) that can be adapted (e.g., fine-tuned) to a wide range of downstream tasks”^(14)^. Initially, large-scale language models trained on vast text data collected from the web solved various language-related tasks (information retrieval, text generation, sentiment analysis, chatbots, etc.) with high accuracy ^(15), (16)^. Subsequently, diverse FMs have been developed using various data modalities, such as images, audio, and point cloud data ^(17)^. According to the “AI Foundation Models: Update paper”^(18)^ by the UK government’s Competition and Markets Authority updated in April 2024, giant AI companies like Meta, Google, and OpenAI are investing millions of dollars to acquire developers. In healthcare, the number of papers related to medical foundation models has exponentially increased from 2018 to February 2024, reflecting the growing expectations and interest of healthcare professionals in applying FMs in clinical practice. While there were only a few papers in 2018, >120 papers were published in 2023 alone ^(19)^.

### (b) Foundation and nonfoundation models

The differences between FMs and non-FMs (deep learning models) are summarized in Table 1 ^(13)^. Generally, the more complex the model, the more diverse relationships it can capture (expressiveness), and the more data, the better the model performance (scalability) ^(20)^. FMs possess superior expressiveness and scalability based on large models, training data, and parallelizable training methods. A common feature of FMs and non-FMs is the need to obtain and utilize the embeddings generated by the model while making inferences from input data. Embeddings represent the features of the input data as short vectors (or small tensors), and the quality of these embeddings remarkably affects the model accuracy during downstream tasks.

**Table 1.** Comparison between Foundation Models and Nonfoundation Models (Adapted from Table 1 in Reference [13]^1^).

Characteristics	Foundation model	Nonfoundation model (deep learning model)
Model architecture	(Mainly) Transformer	Convolutional neural network
Model size	Vary large	Medium to large
Model applicable task	Many	Single
Performance on adapted tasks	State-of-the-art (SOTA)	High to SOTA
Performance on untrained tasks	Medium to high	Low
Data amount for model training	Very large	Medium to large
Using labeled data for model training?	No	Yes

^1^Reference [13] was published under the CC BY 4.0 license.

### (c) Applications of foundation models in healthcare

FMs in healthcare can be broadly categorized into four types based on the data modality they handle ^(19), (21)^:

i) Language Foundation Models for Natural Language Processing (NLP) in Healthcare

Examples of usage: medical report generation, education for medical students/residents, patient self-diagnosis, and mental health support.

ii) Vision Foundation Models (VFMs) for Image Data

Examples of usage: image diagnosis and similar case retrieval, prognosis prediction for specific diseases, and surgical assistance through detection of critical structures and lesions.

iii) Bioinformatics Foundation Models for Omics Data Such as DNA, RNA, and Protein Data

Examples of usage: sequence, interaction, structural, and functional analyses, protein sequence generation, drug response and sensitivity prediction, disease risk prediction, and drug perturbation effect prediction.

iv) Multimodal Foundation Models (MFMs) Integrating Multiple Modalities Such as Language, Image, and Bioinformatics

Examples of usage: comprehensive diagnostic support based on multiple data modalities, medical image report generation, cross-search between text descriptions and chemical structures, promotion of research through dialog with models harboring biological and medical knowledge, and advice on diagnosis and treatment based on patient inquiries and images.

## 3. Introduction for Histopathology Foundation Models

### (a) Need for foundation models in pathology

Representative tasks in computational pathology include patient prognosis prediction, biomarker prediction, cancer and tissue subtype classification, cancer grading, diagnostic prediction, and immunohistochemical staining intensity scoring (Section 1(b)). The development and clinical application of pathology FMs are desired for two reasons:

i) Annotation Cost: Before promoting FM development, the approach to handling individual tasks involved creating supervised learning models based on data annotated by pathologists, which required substantial time and practical effort from pathologists for each disease, organ type, and task type ^(22)^. The average salary of a pathologist is $149 h^−1^ (https://www.salary.com/research/salary/alternate/pathologist-hourly-wages), and assuming 5 min per slide, the annotation cost per pathology slide is approximately $12. The cost of annotation and increasing working hours can be a burden for clinical pathologists ^(22), (23)^. Furthermore, the quality of annotation determines the trained model performance, requiring pathologists to conduct high-quality annotation, thereby adding to their burden and responsibility and becoming a bottleneck in the model development of individual tasks. Model development utilizing vast amounts of pathology image data with minimal or simple annotations per case is crucial.

ii) Lack of Public Datasets: At present, approximately 100 publicly available pathology image datasets ^(24)^ are accessible to model developers, most of which include hundreds to tens of thousands of WSIs. However, the diseases and organ types included in each dataset are limited, and the data quality varies. TCGA―the largest public dataset―including pathology images, contains tens of thousands of WSIs; however, it is limited to 32 cancer types, making it challenging to cover the diverse diseases encountered in clinical practice.

### (b) Examples of pathology foundation models

As of June 2024, more than 10 FMs specifically for pathology images were reported. The origin and scale of datasets, tissue types included, and learning methods vary for each FM. Many models are publicly available and usable for downstream tasks, although some are only available for limited use or are not publicly released (Table 2) ^(10), (22), (23), (25), (26), (27), (28), (29), (30), (31), (32), (33)^.

**Table 2.** List of Pathology Foundation Models with Published Papers, Including Preprints, between October 2022 and June 2024.

Foundation model name	CTransPath	(Lunit)*	PLIP	Virchow	UNI	CONCH	PRISM	Prov-GigaPath	TANGLE	RudolphV
Publication date	2022	2022	2023	2024	2024	2024	2024	2024	2024	2024
Journal name	Medical Image Analysis	arxiv	Nature Medicine	Nature Medicine	Nature Medicine	Nature Medicine	arxiv	Nature	CVPR2024	arxiv
Pretraining dataset name	TCGA	TCGA	OpenPath	MSKCC	Mass-100K	Educational sources	MSKCC)	Dataset from Providence	TCGA	Dataset from over 15 different laboratories across the EU and US
	PAIP	TULIP			Mass-1K	PubMed Central Open Access Dataset			TG-GATEs	TCGA
					Mass-22K					
Number of GPUs/type used for training	48/NVIDIA V100 GPUs	64/NVIDIA V100 GPUs	Not specified	-/NVIDIA A100 GPUs	32/NVIDIA A100 GPUs	8/NVIDIA A100 GPUs	16/NVIDIA V100 GPUs	16 nodes × 4/NVIDIA A100 GPUs	8/NVIDIA A100 GPUs	16/NVIDIA A100 GPUs
Embedding level (patch/slide)	Patch	Patch	Patch	Patch	Patch	Patch	Slide	Slide	Slide	Patch
Number of WSIs	29,763 (TCGA)	20,994 (TCGA)	Not specified	1,488,550	100,426	Not specified	587,196	171,189	2,074 (TCGA)	133,998
	2,457 (PAIP)	15,672 (TULIP)							6,597 (TG-GATEs)	
Number of patch images (M)	15	32.6	0	2,000	100	1	Not specified	1,300	15	1,200
Number of patients	Not specified	Not specified	Not specified	119,629	Not specified	Not specified	195,344	>30,000	>1,864	34,103
More than 10 types of organs in the dataset?	Yes	Yes	Yes	Yes	Yes	Yes	Yes	Yes	No	Yes
Staining types (H&E/H&E + others)	Not specified	H&E	H&E + others	H&E	H&E	H&E + others	H&E	H&E + others	Not specified	H&E + others
FFPE/frozen	FFPE, frozen	Not specified	Not specified	FFPE	FFPE	Not specified	Not specified	Not specified	Not specified	FFPE, Frozen
VFM/MFM	VFM	VFM	MFM	VFM	VFM	MFM	MFM	MFM	MFM	VFM
Model publicly available?	Yes	Yes	Yes	Yes	Yes	Yes	Yes	Yes	No	Yes
Reference number	33	25	28	27	28	30	31	22	10	32

Because of space constraints, not all models are included in this table. For a complete list, please refer to Supplementary Table 1. ^＊^When the model name is not specified in the original paper, the first author’s name is shown.

Herein, we overviewed these pathological FMs by focusing on the following: a) training datasets, modalities, and embedding levels; b) examples of downstream task usage; c) status of public availability; and d) performance comparison between models.

#### a) Training datasets, modalities, and embedding levels

i) Dataset Origin and Scale: Each model is pretrained on a public dataset (e.g., TCGA, PubMed Central Open Access Dataset [https://ncbi.nlm.nih.gov/pmc/tools/openftlist/], TG-GATEs) ^(34)^ or a proprietary clinical pathological image dataset collected from single or multiple institutions). For example, the Virchow ^(27)^ model utilizes a dataset of 1,488,550 WSIs from 119,629 patients stored at Memorial Sloan Kettering Cancer Center and is currently the largest dataset of human pathological images.

ii) Organ and Tissue Types: The organs and tissue types, in addition to their ratios, included in datasets are influenced by various factors, such as whether the dataset includes biopsy/surgical specimen WSIs, specific public datasets, or case accumulation trends of the data collection medical facilities. Although identifying consistent trends is difficult, most datasets used to train FMs include images from >10 different organs. Some models, such as Virchow, UNI ^(29)^, TANGLE ^(10)^, and RudolphV ^(32)^, are explicitly trained on datasets that include normal tissues; however, it is not specified whether other models include only tumor datasets or also normal tissue images. To our knowledge, no experiment has examined the extent to which including normal tissues parallel to tumor tissues in FM training contributes to improved model performance in downstream tasks. Chen et al. ^(29)^ demonstrated that training on a combination of lab-derived datasets and a public dataset, composed of only normal tissue images, resulted in better performance than that on lab-derived datasets alone for the 43-class cancer-type classification task. However, this improvement cannot be conclusively attributed to the mere increase in image count or the inclusion of normal tissue images.

iii) H&E vs. H&E + Other Stains: Although many pathological FMs are trained using datasets with solely hematoxylin and eosin (H&E)-stained images, some models like CONCH and RudolphV include immunostained and/or specially stained images. CONCH conducted comparative experiments by evaluating models trained with only H&E-stained images compared with models trained with other stain types included. The results indicated that models incorporating various stains performed better on most tasks, including tumor subtyping and grading, image-to-text retrieval, and text-to-image retrieval (8/13). However, in some classification tasks, models trained on only H&E-stained images either outperformed or showed marginally lower performance than models with additional stains. This indicates that incorporating diverse stains does not necessarily yield the best results.

iv) VFM/MFM: Many pathological FMs are VFMs; meanwhile, models like PLIP ^(28)^, CONCH, PRISM ^(31)^, Prov-GigaPath ^(22)^, and TANGLE ^(10)^ are MFMs. They handle pathological images and text (e.g., clinical pathology reports, social media, educational sources, and PubMed Central Open Access Dataset captions) and bulk transcriptome data in the case of TANGLE. These MFMs acquire equivalence between paired image and other modality data, enabling model application to tasks such as image-to-text retrieval, text-to-image retrieval, report generation, and gene expression analysis within images.

v) Embedding Levels from Models: WSIs are high-resolution virtual slide images of entire-stained glass slide specimens that often exceed several gigabytes in data size. WSIs have a pyramid structure with multiple layers of images at different magnifications, thereby allowing a comprehensive observation of the entire specimen slide at any zoom level. Memory constraints make it challenging to handle WSIs as whole images during model training; thus, they are typically divided into small patches (tiles) of a few hundred pixels. Therefore, some models output embeddings for each patch, whereas others provide WSI-level embeddings. Models like PRISM, Prov-GigaPath, and TANGLE output WSI-level embeddings, whereas others output patch-level embeddings. Aggregating patch-level embeddings to obtain WSI-level embeddings is possible, and the efficacy of each embedding level for different tasks remains debatable. Prov-GigaPath demonstrated statistically significant improvements over previous patch-level embedding models across 16 tasks, including classification and image-to-text search. However, https://github.com/mahmoodlab/UNI indicates that UNI and CONCH outperformed Prov-GigaPath in four of five classification tasks, including tumor grade classification and immunohistochemical protein expression intensity scoring.

#### b) Examples of downstream tasks

**Figure 1.**
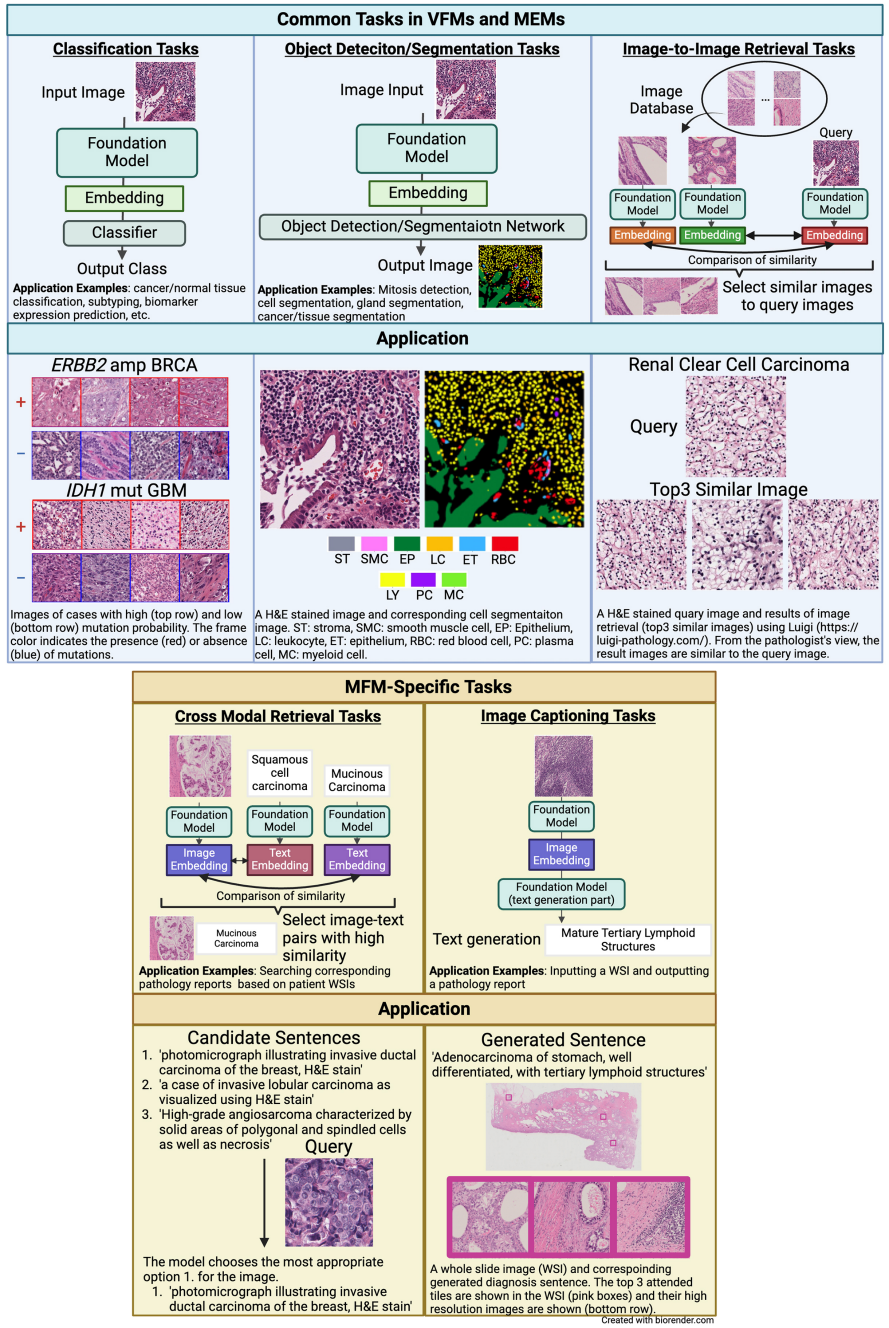
Task-specific schematic of the pathology foundation models (FMs). The figure illustrates five tasks in which pathology FM are applied, including the application example for each task. These include common tasks, such as classification, object detection/segmentation, and image-to-image retrieval, and specific tasks such as cross-modal retrieval and image captioning. The figure highlights the versatility and potential of the pathology FMs in various diagnostic and research contexts.

FMs are applicable to various downstream tasks, with appropriate accuracy verification conducted posttraining. The tasks handled by VFMs/MEMs in healthcare are described in Section 2(c), in which we focus on pathological images and list contents by task category (Figure 1). The tasks mentioned herein are based on those listed in FM papers ^(10), (22), (23), (25), (26), (27), (28), (29), (30), (31), (32), (33)^.

Common Tasks: Predominantly, classification tasks are applicable to VFMs and MFMs, with fewer object detection, segmentation, and search tasks. VFMs such as Lunit ^(25)^, UNI, RandolphV, and CTransPath ^(33)^ have been evaluated for these minor tasks. Reportedly, the SegPath dataset, which we previously published, is used as a benchmark for segmentation tasks. The SegPath dataset is a large-scale dataset comprising >1,500 pairs of H&E- and immunohistochemically-stained images ^(35)^.

i) Classification Tasks: This task involves predicting which category a given image datum (patch or WSI) belongs to. Examples include disease detection, cancer detection, cancer/normal tissue classification, subtyping, disease diagnosis, rare cancer diagnosis, patient prognosis prediction, cancer recurrence prediction, cancer metastasis prediction, biomarker expression prediction (microsatellite instability, driver mutation, and tumor mutation burden), treatment outcome prediction, and immunohistochemical image protein expression intensity scoring.

ii) Object Detection/Segmentation Tasks: This task involves detecting specific structures in images or predicting the category of each pixel. Examples include mitosis detection, cell segmentation in H&E and immunohistochemical images, gland segmentation, cancer segmentation, and tissue segmentation.

iii) Image-to-Image Retrieval Tasks: This task searches for images with high similarity to a query image.

MFM-specific Tasks:

i) Cross-Modal Retrieval Tasks: This is done using an image or text query to search for corresponding text or image pairs.

Examples include retrieving corresponding pathology reports from a database based on patient WSIs.

ii) Image Captioning Tasks: This task generates summary text for an image.

Examples of such inputs include inputting a WSI and outputting a pathology report.

#### c) Public availability status

Most FMs are open access, allowing users to apply them to downstream tasks. However, some models (e.g., CONCH) have limited task availability compared to their original papers or are not open access (e.g., Virchow, PRISM, and RudolphV). The usage instructions for available models are typically described on their respective GitHub (a service for sharing and managing program source code online) pages referenced in the original papers.

#### d) Performance comparison between models

Zeng et al. ^(36)^ conducted comparative accuracy experiments for CTransPath, UNI, Virchow, and Prov-GigaPath across 20 tasks in disease detection, biomarker prediction, and treatment outcome prediction. In disease detection tasks, all four models achieved comparable performances. However, in the biomarker and treatment outcome prediction tasks, UNI and Prov-GigaPath consistently demonstrated performance equal to or exceeding that of the other models. For biomarker prediction in the lung tissue, UNI and Prov-GigaPath outperformed the other models. The authors attributed these results to the higher representation of the lung tissue in the pretraining datasets of UNI and Prov-GigaPath, suggesting that the proportion of relevant tissue in the training dataset could enhance the representational power of the model for that tissue type.

## 4. Issues and Future Directions of Foundation Models

Thus far, we have outlined the development of digital pathology and FMs, their applications in healthcare, the introduction of pathological FMs, and specific examples of their usage. This section focuses on the future implications of FM-related research and discusses potential issues users might face while employing FMs in clinical AI applications.

### (a) Hardware requirements

Many FMs require specific hardware because of their immense complexity and scale. Although these models demonstrate remarkable performance, their training and inference processes often require substantial computational resources. For example, running Prov-GigaPath requires high-end Graphics Processing Units (GPUs), such as NVIDIA A100 ^(37)^. Hardware investment required for these models can be challenging for resource-constrained medical institutions. Therefore, balancing model performance with hardware feasibility is critical while implementing these models clinically. Future research should optimize these models to operate on less resource-intensive hardware, thereby making them accessible to several medical facilities.

### (b) Transition to multimodal AI assistants and generalist medical AI

At present, VFMs are the most developed pathological FMs. However, various MFMs have been developed, focusing on combining language and visual modalities to produce pathology diagnostic reports from image inputs or molecular biology and visual modalities to identify notable genes in model-driven pathology diagnoses. These MFMs are mainstream in pathology and across healthcare ^(19)^. The current self-supervised learning methods are not universally generalizable across all modalities and often require tailoring per specific modalities ^(21)^. This limitation means that integrating more modalities into FMs in real-world clinical settings is not feasible. In 2023, Lu et al. ^(38)^ developed a dialog-based pathological AI assistant capable of prompt engineering, which improved task accuracy by interactively providing appropriate questions and instructions, was built on previously developed pathological MFMs by their group and supported diagnostic assistance by describing histological findings and suggesting additional tests through interactions with pathologists. Recently, the concept of generalist medical artificial intelligence (GMAI) ^(39)^ has been proposed, utilizing vast and diverse data types―including images, electronic health records, test results, genomics, graphs, and medical texts―to perform various tasks across different medical specialties. The development of AI assistants integrating FMs from domains beyond pathology will possibly drive comprehensive medical AI research and development toward realizing GMAI.

### (c) Hallucinations and interpretability

Models generate embeddings through probabilistic processes instead of truly understanding the logical meaning of data ^(40)^. Consequently, FMs might generate nonexistent information (fabricated citations, information not inferable from input data, etc.) during generative tasks such as pathology report generation (hallucinations) ^(41), (42)^. Users must recognize these potential hallucinations. Furthermore, the inference processes of large-scale FMs with hundreds of millions to trillions of parameters are incredibly complex and are often described as the “black box” nature of AI. Unlike traditional medical devices that are typically transparent and logical, this characteristic makes it difficult for people to trust the algorithm results ^(43)^. Thus, the field of “explainable AI ^(44)^” is advancing, developing techniques to enhance human understanding of how AI models reach their outputs.

### (d) Potential for domain shift and information updates

Pathological specimens from Japan and overseas may show remarkable color and texture differences, even with the same staining. Because all publicly available pathological FMs were developed using datasets from overseas facilities, they might not generalize to Japanese specimens (WSIs) because of color and texture distribution (domain shift ^(45)^). Various methods have been developed to efficiently standardize color tones and perform color augmentation. Previously, we created a large-scale pathological image dataset, PLISM ^(46)^, which includes tissues stained with H&E in various color tones that facilitate color augmentation. Moreover, there are potential issues pertaining to handling and processing of tissue samples, and the patient specimen data used for training FMs may have inherent biases concerning gender, race, age, and sampling methods. If the sample appearance, such as color tone, or the social and sampling method aspects of the to-be predicted data significantly differ from the training data, the analysis and prediction accuracy of FMs may decrease. Further, the lack of standardized evaluation metrics and benchmarks complicates the validation process, thereby making it difficult for healthcare professionals to accurately assess the performance of these FMs. Additionally, the data used to train these large models, including PubMed and textbook knowledge, can quickly become outdated because of the rapid advancements in medicine, necessitating regular updates to prevent inaccuracies. However, retraining these large-scale models with new data is costly. Researchers aim to improve these model architectures using retrieval-augmented generation (RAG) ^(15)^, which allows models to reference external databases while generating responses, consequently enhancing accuracy and explainability.

### (e) Concerns about the clinical implementation of foundation models

Despite the rapid development of FMs, their validation in real clinical settings remains insufficient, preventing their clinical application. Similar to other AI models, FMs are regulated as medical devices by the US Food and Drug Administration under a uniform software category developed for specific-use cases ^(47)^. The World Health Organization released AI ethics and governance guidance for MFMs in 2024, including recommendations in development and deployment for governments and developers ^(48)^. The guidance outlines more than 40 considerable recommendations for governments, technology companies, and healthcare providers to ensure the appropriate use of MFMs for promoting and protecting the population health. However, the current regulatory environment is inadequate for the clinical safety and effective deployment of FMs. Only a few approved AI models have been tested in randomized controlled trials and none have involved FMs ^(49)^. The lack of transparency in trial reports and evaluated use cases is a considerable issue. The Standard Protocol Items: Recommendations for Interventional Trials―AI and consolidated standards of reporting trials―AI guidelines ^(50)^, which were assessed by an international multistakeholder group from specific fields, such as healthcare, statistics, computer science, law, and ethics, in a two-stage Delphi survey and agreed upon in a 2-day consensus meeting, have been formulated to improve standardization and transparency in clinical trials involving AI; however, most published randomized controlled trials that use AI technology have not strictly followed these established reporting standards ^(51)^. Users must be aware of the insufficient algorithm validation of FMs in real clinical settings. At present, there are no pathological FMs approved by the FDA for commercial use, and utilizing pathological FMs remains confined to academic research ^(52)^.

## 5. Conclusion

Advances in digital pathology have expanded the utilization of WSIs, leading to active research and development of related AI technologies. Particularly, the emergence of FMs has broadened the scope of AI applications in medicine, allowing the development of AI models capable of handling various tasks via training on diverse data, such as images, texts, and omics information. In the future, AI-based medical practice using FM-based AI assistants and GMAI will potentially be realized in pathology and real clinical settings, promoting efforts toward precision and personalized medicine. Although medical AI is an extremely useful tool in practice, properly understanding the effectiveness, considerations, and potential issues of using AI centered on FMs to benefit patients is crucial for medical professionals, who are the primary users of FMs.

## Article Information

### Conflicts of Interest

None

### Sources of Funding

This work was supported by the AMED Practical Research for Innovative Cancer Control grant number JP 24ck0106873 and JP 24ck0106904 to S.I., JSPS KAKENHI Grant-in-Aid for Scientific Research (S) grant number 22H04990 to S.I., and JSPS KAKENHI Grant-in-Aid for Scientific Research (B) grant number 21H03836 to D.K.

### Author Contributions

M.O. contributed to the search of previous publications and wrote the manuscript draft. D.K. and S.I. reviewed the manuscript critically.

### Approval by Institutional Review Board (IRB)

Not applicable.

## Supplement

Supplementary Table 1 List of pathology foundation models with published papers, including preprints, between October 2022 and June 2024.
*When the model name is not specified in the original paper, the first author’s name is shown.
