# Supplementary material for: Pathology Foundation Models
Source: JMA J. 2024 Dec 20;8(1):121–30. doi: 10.31662/jmaj.2024-0206 (PMC11799676; doi:10.31662/jmaj.2024-0206)
Supplement: Supplementary Table 1 — List of pathology foundation models with published papers, including preprints, between October 2022 and June 2024. *When the model name is not specified in the original paper, the first author’s name is shown. [file 2433-3298-8-1-0121-s001.pdf]

Foundation Model Name	CTransPath	(Lunit)*	Phikon	PLIP	REMEDIS	Virchow	UNI	CONCH	PRISM	Prov-GigaPath	TANGLE	RudolphV
Publication Date	2022	2022	2023	2023	2023	2023	2024	2024	2024	2024	2024	2024
Journal Name	Medical Image Analysis	arxiv	arxiv	Nature Medicine	Nature biomedical engineering	arxiv	Nature Medicine	Nature Medicine	arxiv	Nature	CVPR2024	arxiv
Pretraining Dataset	TCGA<br>PAIP	TCGA<br>TULIP	PanCancer40M<br>TCGA-COAD	OpenPath	JFT-300M<br>CAMELYON16<br>TCGA<br>Colorectal tissue slides from the Institute of Pathology and the Biobank at the Medical University of Graz	Dataset from Memorial Sloan Kettering Cancer Center (MSKCC)	Mass-100K<br>Mass-1K<br>Mass-22K	Educational sources<br>PubMed Central Open Access Dataset	Dataset from Memorial Sloan Kettering Cancer Center (MSKCC)	Dataset from Providence	TCGA<br>TG-GATEs	Dataset from over 15 different laboratories across the EU and US<br>TCGA
Number of GPUs/Type Used for Training	48/ NVIDIA V100 GPUs	64/ NVIDIA V100 GPUs	16-64/ NVIDIA V100 GPUs	Not Specified	16–256/ Google Cloud TPU cores	–/NVIDIA A100 GPUs	32/ NVIDIA A100 GPUs	8/ NVIDIA A100 GPUs	16/ NVIDIA V100 GPUs	16 nodes × 4/ NVIDIA A100 GPUs	8/ NVIDIA A100 GPUs	16/ NVIDIA A100 GPUs
Embedding Level (patch/slide)	patch	patch	patch	patch	patch	patch	patch	patch	slide	slide	slide	patch
Number of WSIs	29,763(TCGA)<br>2,457(PAIP)	20,994(TCGA)<br>15,672(TULIP)	6,093(PanCancer40M)<br>441(TCGA-COAD)	Not Specified	29,018(TCGA)	1,488,550	100,426	Not Specified	587,196	171,189	2,074(TCGA)<br>6,597(TG-GATEs)	133,998
Number of Patch Images(M)	15	32.6	43	0	50	2,000	100	1	Not Specified	1,300	15	1,200
Number of Patients	Not specified	Not specified	5,558	Not specified	10,705	119,629	Not specified	Not specified	195,344	>30,000	>1,864	34,103
More than 10 types of organs in the dataset?	Yes	Yes	Yes	Yes	Yes	Yes	Yes	Yes	Yes	Yes	No	Yes
Staining Types (H&E/H&E+Others)	Not specified	H&E	H&E	H&E+Others	Not Specified	H&E	H&E	H&E+Others	H&E	H&E+Others	Not Specified	H&E+Others
FFPE/frozen	FFPE, frozen	Not Specified	FFPE	Not Specified	Not Specified	FFPE	FFPE	Not Specified	Not Specified	Not Specified	Not Specified	FFPE, Frozen
VFM/MFM	VFM	VFM	VFM	MFM	VFM	VFM	VFM	MFM	MFM	MFM	MFM	VFM
Model Publicly Available?	Yes	Yes	Yes	Yes	Yes	Yes	Yes	Yes	Yes	Yes	No	Yes
Reference Number	33	25	26	28	23	27	29	30	31	22	10	32
